# Effectiveness of mobilization with movement in patients operated for distal radius fracture: a single-blinded, randomized controlled study

**DOI:** 10.1590/1806-9282.20241190

**Published:** 2024-12-02

**Authors:** Levent Horoz, Basak Cigdem-Karacay, Ismail Ceylan, Halil Alkan

**Affiliations:** 1 Kirsehir Ahi Evran University, Faculty of Medicine, Department of Orthopedics and Traumatology – Kırşehir, Turkey.; 2 Kirsehir Ahi Evran University, Faculty of Medicine, Department of Physical Medicine and Rehabilitation – Kırşehir, Turkey.; 3 Kirsehir Ahi Evran University, School of Physiotherapy and Rehabilitation – Kırşehir, Turkey.; 4 Mus Alparslan University, Faculty of Health Sciences, Department of Physiotherapy and Rehabilitation – Muş, Turkey.

**Keywords:** Mobilization, Exercise therapy, Wrist fractures, Range of motion, Edema, Hand

## Abstract

**OBJECTIVE:**

BACKGROUND: There are limited data on the use of Mulligan's mobilization with movement technique in patients who underwent surgery for distal radius fracture. AIMS: This study aimed to evaluate the effectiveness of adding Mulligan's mobilization with movement to the conventional exercise program for those who underwent open reduction and volar plate application due to distal radius fracture.

**METHODS:**

This randomized controlled, single-blind study was conducted with 53 patients who had been operated on for distal radius fracture. The patients were divided into two groups, the mobilization with movement group and the control group. Patients in the mobilization with movement group were mobilized using Mulligan's mobilization with movement technique in addition to the rehabilitation program used in the control group. The degree of volar inclination and radial inclination were recorded. Radial height was measured in millimeters. Ulnar variance was recorded. Perimeter was measured using the Figure of Eight method. The range of motion of the joint was measured by goniometry. Hand grip strength was measured with Jamar® hand dynamometer, and pinch grip was measured with a pinch meter. The Patient-Rated Wrist Evaluation questionnaire was used to assess functionality.

**RESULTS:**

There is no statistically significant difference identified between the groups (p>0.05). The intra-group changes in the data of the groups were found to be statistically significant in visual analog scale, range of motion, pinch grip, hand grip, and Patient-Rated Wrist Evaluation parameters (p<0.05). There was a statistically significant difference between the mobilization with movement group and the control group for pronation value and hand grip strength value in measurements (p<0.05).

**CONCLUSIONS:**

XThe mobilization with movement had no additive effect on parameters other than hand grip strength and pronation.

## INTRODUCTION

The incidence of distal radius fractures (DRF) is anticipated to increase annually, mainly because of the extension in life expectancy and osteoporosis^1,2^. If DRFs are not treated properly, they may result in restricted daily functioning and permanent disabilities. Therefore, it is important to administer effective treatment^3,4^. Open reduction and volar plate application are frequently preferred surgical methods in the surgical treatment of DRFs^5^. Rehabilitation is advisable following surgical intervention for DRF^6^.

Mobilization with movement (MWM) is a manual therapy method. Mulligan's MWM technique is particularly favored for peripheral joints because it effectively realigns the joint and restores its normal tracking, thereby reducing pain^7–9^. Mulligan's MWM technique is applied continuously by an experienced physiotherapist^10^. It allows a more painless mobilization by restoring the biomechanical harmony on the joint surfaces^11^.

It has been reported that adding MWM applied with Mulligan's MWM technique to exercise will improve joint range of motion in conservatively managed patients with DRF^9^. A meta-analysis conducted recently, which focused on the effect of adding manual therapy into clinical studies for DRF rehabilitation, indicated that mobilization could possibly impact function and edema but not joint range of motion^12^. Further investigation is necessary in this field, employing studies conducted on a more comprehensive sample size and containing detailed data regarding fracture classification and treatment modalities^12,13^. To the best of the author's knowledge, only one study has evaluated the efficacy of Mulligan's MWM technique in patients who have undergone open reduction and volar plating after DRF^13^. This study aimed to evaluate the effectiveness of adding Mulligan's MWM technique to the conventional exercise program on pain, joint range of motion, and muscle strength in patients who underwent open reduction and volar plate application due to DRF.

## METHODS

### Study population and design

This randomized controlled, single-blind study was conducted with 53 patients at the University Hospital Orthopedics and Physical Medicine and Rehabilitation clinics between August 2023 and November 2023.

Patients above 18 years of age who underwent open reduction and volar plate surgery due to DRF with direct X-ray and CT were evaluated. Patients who did not develop any major postoperative complications (such as neurovascular injury and hematoma) were included in the study. Exclusion criteria of the study were the presence of polytrauma, surgical intervention other than volar plate, previous extremity-related surgery history, injury in more than one anatomical region of the relevant extremity, and limitation (such as hemiplegia and contracture) in the relevant extremity.

### Randomization and blinding

The patients were divided into two groups: the MWM group and the control group using the 1:1 randomization method. Randomization was performed by the same investigator (Z) using a computer-generated list of random numbers on Excel© 2019 software. The doctor who made the evaluations for blinding (Y) was unaware of the groups.

### Outcome measures

Outcome measures were assessed at baseline and at week four. Pain intensity, grip strength, pinch strength, wrist joint range of motion, edema, and upper extremity functionality were evaluated for all patients. Descriptive features of gender, age, mass, height, dominant side, affected side, and smoking habits were documented during the initial evaluation. Radiographic evaluation was performed to examine the patients’ fracture reduction parameters.

### Interventions

#### Control group

The rehabilitation program was created by a physical therapy specialist (Y), orthopedic surgeon (X), and physiotherapist (Z) before the study. The splint was removed in the second week, according to the rehabilitation program. Forearm, wrist, and finger passive range of motion (ROM) stretching and active assisted ROM stretching exercises were performed between 2 and 4 weeks. Isometric strengthening, isotonic strengthening, and strengthening exercises with light-medium hard play dough and the Digi-Flex hand exerciser (IMC Products Corp., Hicksville, New York) were performed between 4 and 8 weeks. Additionally, transcutaneous electrical nerve stimulation TENS Intellect® Advanced (Chattanooga, Mouguerre, France) was applied to the patients using the conventional method. The rehabilitation program was individualized according to the patients. The same rehabilitation program was applied to all patients who participated in the study.

#### Mobilization with movement group

Patients in this group were mobilized using Mulligan's MWM technique in addition to the rehabilitation program used in the control group. The MWM technique was performed on all patients by a certified physiotherapist with 5 years of experience in this practice. Movement mobilization is a method in which active physiological end-of-range movement applied by the patient and continuous accessory mobilization applied by the physiotherapist are applied simultaneously.

All of these techniques were performed in three sets of 10 repetitions. Each of these techniques is described in detail in a textbook on MWM^14^. The interventions were performed 3 days a week for 4 weeks, with sessions lasting an average of 30–40 min. All patients in the study were also taught how to position the affected side at rest, how to avoid overloading the hand, and how to perform functional activities in the safest way.

### Sample size

Grip strength was measured using version 3.1.9.4 of the G-Power program (Heinrich-Heine-Universitat Düsseldorf, Germany) to define the study sample^15^. To achieve 80% power (1 - β error probability) with an α error level probability of 0.05, we performed repeated measures analysis of variance (ANOVA) both within and between interactions, using a mean effect size of 0.30 to account for the two groups and using two measurements for the primary outcome. A total of 47 participants were recruited into the study to account for the 15% dropout rate and to increase the statistical power of the results.

### Statistical analysis

Statistical analyses were performed using the SPSS version 25 software. Visual (histogram and probability plot) and analytical (Shapiro-Wilk test) methods were used to test the suitability of the variables for normal distribution. Descriptive analyses were given using the mean and standard deviation for normally distributed variables. For nominal variables, numbers and percentages were given. In comparing the measured values of the groups, Student's t-test was used to test the significance of the difference between the two means. The chi-square test (Pearson chi-square) was used to examine the relationship between categorical variables. A two-way analysis of variance (mixed-design repeated-measures ANOVA) was used to evaluate the change over time in the measured variables of the groups and group-time interactions. For statistical significance, the type-1 error level was set at 5%. The statistical analysis of the study was conducted by a statistician (T) blind to the groups.

### Ethics approval and consent to participate

This research has been approved by the IRB of the authors’ affiliated institutions. This study was conducted in accordance with the 1964 Helsinki Declaration, and a written consent form was obtained from all patients. The trial was registered on Clinicaltrials.gov before the first patient was recruited.

## RESULTS

Upon comparing the descriptive characteristics of the groups studied, no statistically significant difference was identified between the groups (p>0.05). The distribution of the groups in terms of descriptive characteristics was similar (Table 1). There were no differences between the two groups in terms of volar inclination degree, radial inclination, radial height, and ulnar variance, which are radiographic fracture healing parameters of the patients (Table 2). According to the mixed design repeated measures ANOVA analysis, the changes over time (intra-group changes) in the numerical measurement data of the groups were found to be statistically significant in visual analog scale (VAS), ROM, pinch grip, hand grip, and Patient-Rated Wrist Evaluation (PRWE) parameters (p<0.05). Comparing the numerical measurement data of the groups over time revealed that there was a statistically significant difference between the MWM group and the control group for pronation value and hand grip strength value in ROM measurements (p<0.05). However, no significant difference was found for the other parameters (p>0.05) (Table 3).

**Table 1:** Comparison of descriptive features of groups.

		MWM group (n=23)		Control group (n=24)		t	p
		X	SD	X	SD		
Age (years)		55.8	8.7	54.6	10.9	0.42	0.670
Height (cm)		166.3	7.2	163.5	9.0	1.15	0.255
Weight (kg)		74.0	12.0	71.6	6.7	0.03	0.061
		**n**	**(%)**	**n**	**(%)**	**X^2^**	**p**
Gender	Female	15	65.2	19	79.2	1.14	0.285
	Male	8	34.8	5	20.8		
Dominant hand	Right	21	91.3	20	83.3	0.67	0.413
	Left	2	8.7	4	16.7		
Operated hand	Right	10	43.5	13	54.2	0.53	0.464
	Left	13	56.5	11	45.8		
Smoking	Right	21	91.3	18	75.0	2.21	0.137
	Left	2	8.7	6	25.0		
AO/OTA Fracture Classification	A type	4	17.3	5	20.8	0.73	0.617
	B type	9	39.3	9	37.6		
	C type	10	43.4	10	41.6		

MWM: mobilization with movement; BMI: body mass index; t: t-test in independent groups; AO/OTA: AO Foundation/Orthopedic Trauma Association; X²: chi-square Analysis, X: mean; SD: standard deviation.

**Table 2:** Postoperative reduction parameters.

	MWM group (n=23)		Control group (n=24)		p-value
	Mean	SD	Mean	SD	
Volar tilt, degree (°)	12.4	1.2	11.9	1.3	0.312
Radial inclination (°)	21.4	1.8	21.7	1.6	0.216
Radial height (mm)	12.1	0.8	11.8	1.1	0.651
	<2 mm	>2 mm	<2 mm	>2 mm	
Ulnar variance (n)	21	2	22	2	0.215

MWM: mobilization with movement; SD: standard deviation.

**Table 3:** Comparison of pre-treatment and post-treatment data of the groups.

			MWM group (n=23)		Control group (n=24)		Time (main effect)	Group* Time (interaction)	Pairwise comparison		η^2^
			Mean	SD	Mean	SD	p	F/p-value	MWM Group (Mean Diff./p)	Control Group (Mean Diff. /p)	
VAS	Rest	Baseline	4.7	2.4	5.5	2.0	**0.000**	0.23/0.631	–	–	0.005
		PT	0.7	0.9	1.8	1.2					
	Activity	Baseline	5.9	2.6	6.1	1.9	**0.000**	0.24/0.622	–	–	0.005
		PT	1.4	1.2	1.3	1.2					
ROM	Flexion	Baseline	38.2	12.9	35.0	15.9	**0.000**	0.18/0.666	–	–	0.004
		PT	64.1	9.4	62.5	9.0					
	Extension	Baseline	36.3	14.4	32.7	11.7	**0.000**	0.08/0.775	–	–	0.002
		PT	56.7	10.2	54.1	7.1					
	Supination	Baseline	49.5	18.4	52.5	11.8	**0.000**	0.01/0.908	–	–	0.001
		PT	76.7	10.0	79.1	8.6					
	Pronation	Baseline	60.6	13.7	78.7	9.8	**0.000**	23.83/**0.000**	23.26/**0.000**	−5.62/**0.031**	0.346
		PT	83.9	6.2	84.3	7.7					
Pinch Grip		Baseline	2.1	0.6	1.5	0.7	**0.000**	0.73/0.396	–	–	0.010
		PT	4.8	1.3	3.7	1.1					
Hand Grip		Baseline	9.7	3.4	11.9	2.8	**0.000**	27.09/**0.000**	11.73/**0.000**	−3.92/**0.000**	0.380
		PT	21.5	5.2	15.8	3.5					
PRWE		Baseline	64.1	23.2	73.0	20.0	**0.000**	2.66/0.110	–	–	0.056
		PT	15.7	11.7	34.5	19.6					
Edema Figure of Eight (cm)		Baseline	2.1	1.2	2.3	1.3			–	–	0.247
		PT	1.2	0.4	1.1	0.6			–	–	0.348

MWM: mobilization with movement; VAS: visual analog scale, PRWE: the Patient-Rated Wrist Evaluation questionnaire; ROM: range of motion; PT: post-treatment; Diff: difference; two-way analysis of variance in repeated measurements (mixed-design repeated-measures ANOVA); SD: standard deviation; **η** ^2^: effect size

* difference from the healthy side. Bold values indicate p<0.001.

## DISCUSSION

According to the results of this study, adding MWM to the exercise program of patients who were operated on with open reduction and volar plate application due to DRF is effective on hand grip strength and wrist joint pronation. However, adding MWM to exercise therapy does not affect pain, compression grip, functionality, and joint flexion, extension, and supination.

In orthopedic rehabilitation, managing pain is just as crucial as addressing edema. The existence of pain can result in avoidance reactions, hinder adherence to exercise regimes, and affect an individual's functionality^16,17^. For this reason, it is reported that early mobilization is necessary for pain and edema control in DRF rehabilitation. In this study, patients were followed up with a plaster splint postoperatively for two weeks, followed by exercise and rehabilitation. It is important to have pain-free movement to improve muscle strength. The Mulligan technique focuses on pain-free mobilization and is an approach that breaks the pain avoidance cycle^11,13^. This approach could enhance the effectiveness of resistance training. Tomruk et al.'s study indicates that MWM, utilizing the Mulligan technique, is an effective rehabilitation method for patients who have undergone DRF surgery, increasing muscle strength^13^. The results of this study corroborate the aforementioned information. The rise in grip strength can be explained by the painless mobilization facilitated using this technique.

In recent years, the literature has seen a rise in studies assessing the efficacy of MWM in rehabilitating DRFs. This intervention has been frequently implemented in patients undergoing conservative treatment. In addition, it has been reported that fracture classification and reduction parameters were not evaluated in the majority of the studies^11^.

Reid et al. conducted a study on conservatively managed patients with DRFs and concluded that incorporating exercise with MWM enhanced joint range of motion; however, it exhibited no impact on muscle strength and functionality^9^. Biswas et al. conducted a study that found that the Mulligan technique applied with MWM did not effectively improve active and passive joint range of motion in patients with DRFs. The study made no distinction between conservative and surgical methods and focused solely on the effectiveness of joint range of motion. Pain and function were not assessed, and the evaluation solely focused on the effectiveness of joint range of motion^18^.

To the best of the authors’ knowledge, there is only one study in the literature evaluating the effectiveness of MWM in patients who have undergone open reduction and volar plating for DRFs. This study by Tomruk et al. reported that the addition of MWM using the Mulligan technique to the exercise program was effective on pain, functionality, and grip strength. Fracture classification and reducing parameters were also considered. The results of our study also support the effectiveness of MWM on muscle strength, but no additional effect on pain or functionality was observed in this study. Tomruk et al. reported that they started rehabilitation on day 7. In this study, rehabilitation started after day 14^13^. We thought that the effect on pain and function might be related to the early start of mobilization.

The short follow-up period was the main limitation of this study. Only post-treatment efficacy was evaluated. However, the fact that all patients in the study were operated on by the same trauma-experienced orthopedic surgeon and that reduction parameters and fracture classification were included in the study allowed a homogeneous selection of the sample. In addition, the single-blind design of the study and the fact that all applications were performed by the same physiotherapist experienced in manipulation and hand surgery are the strengths of this study.

## CONCLUSION

Although MWM is a technique used in orthopedic rehabilitation, there is a paucity of data in the literature regarding its use in clinical practice, and studies are inconsistent. In this study, we observed that MWM had no additive effect on parameters other than hand grip strength and pronation. Further well-designed studies are required to incorporate MWM into the rehabilitation of patients with DRFs.
